# Cellulose nanocrystals formation leveraging synergy between lytic polysaccharide monooxygenase and endoglucanase

**DOI:** 10.1007/s00449-026-03420-9

**Published:** 2026-09-18

**Authors:** Lauren E. Lynn, William Hart-Cooper, William Orts, Tina Jeoh

**Affiliations:** 1 Biological and Agricultural Engineering, University of California Davis, Davis, CA USA; 2 Bioproducts Research Unit, Western Regional Research Center, USDA-ARS, Albany, CA USA

**Keywords:** Cellulose nanocrystals (CNC), Endoglucanase, Lytic polysaccharide monooxygenase (LPMO), Synergy, Biomaterial, Enzyme synergy

## Abstract

Cellulose nanocrystals (CNCs) are crystalline fragments released from cellulose via selective disruption of the fibrillar structure. This study investigated CNC formation by leveraging synergy between a C1/C4-active lytic polysaccharide monooxygenase (LPMO) *Ta*AA9A and glycoside hydrolase family 45 endoglucanase (EG) NsGH45. The LPMO *Ta*AA9A alone could not liberate CNCs from the cellulose substrate, and the endoglucanase NsGH45 alone yielded limited CNC release; their co-incubation, however, exhibited strong synergy, increasing CNC yields by up to 81% relative to additive enzyme action. Varying enzyme ratios in the reaction revealed distinct LPMO: EG ratios to achieve maxima for CNC formation versus soluble sugar release, indicating that the reaction can be tuned to favor CNC production over sugar solubilization. Further, LPMO activity was shown to increase productive cellobiohydrolase binding capacity of residual cellulose, supporting a mechanism whereby LPMO activity increases endoglucanase access to previously inaccessible regions of the cellulose fibrils. Our findings demonstrate that LPMO-endoglucanase synergy can be harnessed and tuned in cellulose deconstruction pathways towards CNC production.

**Supplementary Information:**

The online version contains supplementary material available at https://doi.org/10.1007/s00449-026-03420-9.

## Introduction

Cellulose nanocrystals (CNC) are highly crystalline colloidal nanoparticles with at least one dimension < 1 μm that is derived from breaking down the hierarchical structure of cellulose. Owing to their exceptional mechanical properties, high thermal stability (260˚C), low density, high surface area, and tunable surface chemistry, CNCs have emerged as promising sustainable materials for applications in polymer nanocomposites, rheological modifiers, coatings, and advanced functional materials [1]. Industrial production of CNCs is currently dominated by concentrated mineral acid hydrolysis that preferentially removes amorphous regions of cellulose, liberating recalcitrant and crystalline cellulose nanocrystals. Although high CNC yields can be achieved, acid hydrolysis has drawbacks including degradation of fermentable sugars, and challenges in acid recovery [2]. Technoeconomic analyses have also shown that while acid-based production can achieve relatively low production cost, it is associated with significant energy demand and environmental burdens due to reagent recovery and processing requirements [3]. These limitations have motivated the search for milder, more sustainable production routes, including enzymatic methods.

Enzymatic processing offers a selective and environmentally benign alternative for cellulose deconstruction with improved control over fiber structure and reduced environmental impact, although they typically exhibit lower efficiency and longer processing times and higher costs due to enzyme requirements and reaction durations [4]. Developing strategies to enhance the effectiveness of enzymatic routes is therefore critical to advancing sustainable CNC production. Among cellulases, endoglucanases (EG, EC 3.2.1.4) hydrolyze internal glycosidic bonds, primarily within disordered or amorphous regions of cellulose [5]. Endoglucanases rapidly reduce cellulose fibrils lengths [6], and are thus considered to be well suited to facilitate release of crystalline fragments for CNC production [7, 8]. However, endoglucanase catalyzed CNC production processes suffer from low yields [4].

In contrast to hydrolytic enzymes, lytic polysaccharide monooxygenases (LPMO), catalyze oxidative cleavage of glycosidic bonds through a copper-dependent mechanism [9–11]. Cellulose-active LPMOs feature flat active sites that enable interactions with glycosidic bonds in crystalline cellulose without requiring chain extraction, which allows them to disrupt highly ordered regions of the fibrils [12]. Several studies have shown that LPMO activity increase the hydrolytic efficiency of cellulases by increasing accessibility to productive complexation sites [13, 14]. LPMOs have been demonstrated to act synergistically with endoglucanases to accelerate cellulose saccharification to increase soluble sugar yields [15, 16]. Despite extensive work on LPMO-cellulase synergy for sugar production, its potential relevance to nanocellulose production remains largely unexplored. Here, we examine whether LPMO-cellulase synergy can be leveraged not just to enhance cellulose depolymerization, but to promote and control the formation of cellulose nanocrystals. This represents a critical knowledge gap, as unlike hydrolytic processes targeting complete saccharification, CNC production requires selective deconstruction to preserve crystalline domains while achieving sufficient fibril fragmentation and dispersion. Beyond mechanistic insight, the ability to control the balance between nanocrystal formation and saccharification represents an important lever for bioprocess design. In enzymatic cellulose conversion, product distribution directly impacts downstream processing requirements and process economics. Therefore, understanding how enzyme composition governs this balance may enable rational tuning of enzymatic systems to favor either particulate nanomaterials or soluble sugars, depending on the desired application.

In this study, we investigated leveraging synergy between LPMO and endoglucanase for CNC production, with the goal of directing cellulose deconstruction toward nanomaterial formation rather than complete saccharification. Specifically, a C1/C4-active LPMO from *Thermoascus aurantiacus* (*Ta*AA9A) [10] and a glycoside hydrolase family 45 endoglucanase (NsGH45) were evaluated individually and in combination. NsGH45 is an inverting endoglucanase in the glycoside hydrolase (GH ) family 45. NsGH45 contains a carbohydrate binding module (CBM) and has a molecular weight of 43 kDa. The enzyme structure is a six-stranded ß-barrel with an appended seventh strand. The enzyme active site (length = 50 Å, width = 12 Å, depth = 13 Å) has a long, wide, and open substrate binding groove with seven sub-sites [17, 18]. *Ta*AA9A is a 27.42 kDa thermophilic fungal LPMO from *Thermoascus aurantiacus* that catalyzes oxidative cleavage of cellulose and, to a lesser extent, xylan via a copper-dependent mechanism requiring a reducing agent and O₂ or H₂O₂ [19]. *Ta*AA9A, a Type-3 AA9 enzyme lacking a CBM that produces both C1- and C4-oxidized products, yield aldonic acids and 4-ketoaldoses that enhance cellulose depolymerization. Structural studies show the canonical histidine-brace copper active site and reveal features such as glycosylation and loop variations that may influence substrate binding [20]. *Ta*AA9A is notable for its thermostability, retaining substantial activity up to ~ 70 °C, and measurable kinetics, making it a useful model enzyme.

We quantified CNC yields and soluble sugar production at varying NsGH45:*Ta*AA9A enzyme ratios to determine how enzyme composition can be used to tune product selectivity. Furthermore, to elucidate the mechanistic basis of the synergy, we assessed changes in substrate accessibility through measurements of productive cellulase binding capacities of the residual cellulose. We hypothesized that LPMO activity on crystalline cellulose increases accessibility for endoglucanase action, enabling controlled fragmentation that favors CNC release while minimizing excessive solubilization. The results provide insight into how enzyme synergy can be tuned to direct cellulose deconstruction pathways towards nanomaterial production rather than complete hydrolysis, offering a potential route toward more sustainable CNC manufacturing.

## Experimental section

### Preparation of cellulose substrate

**Fig. 1:**
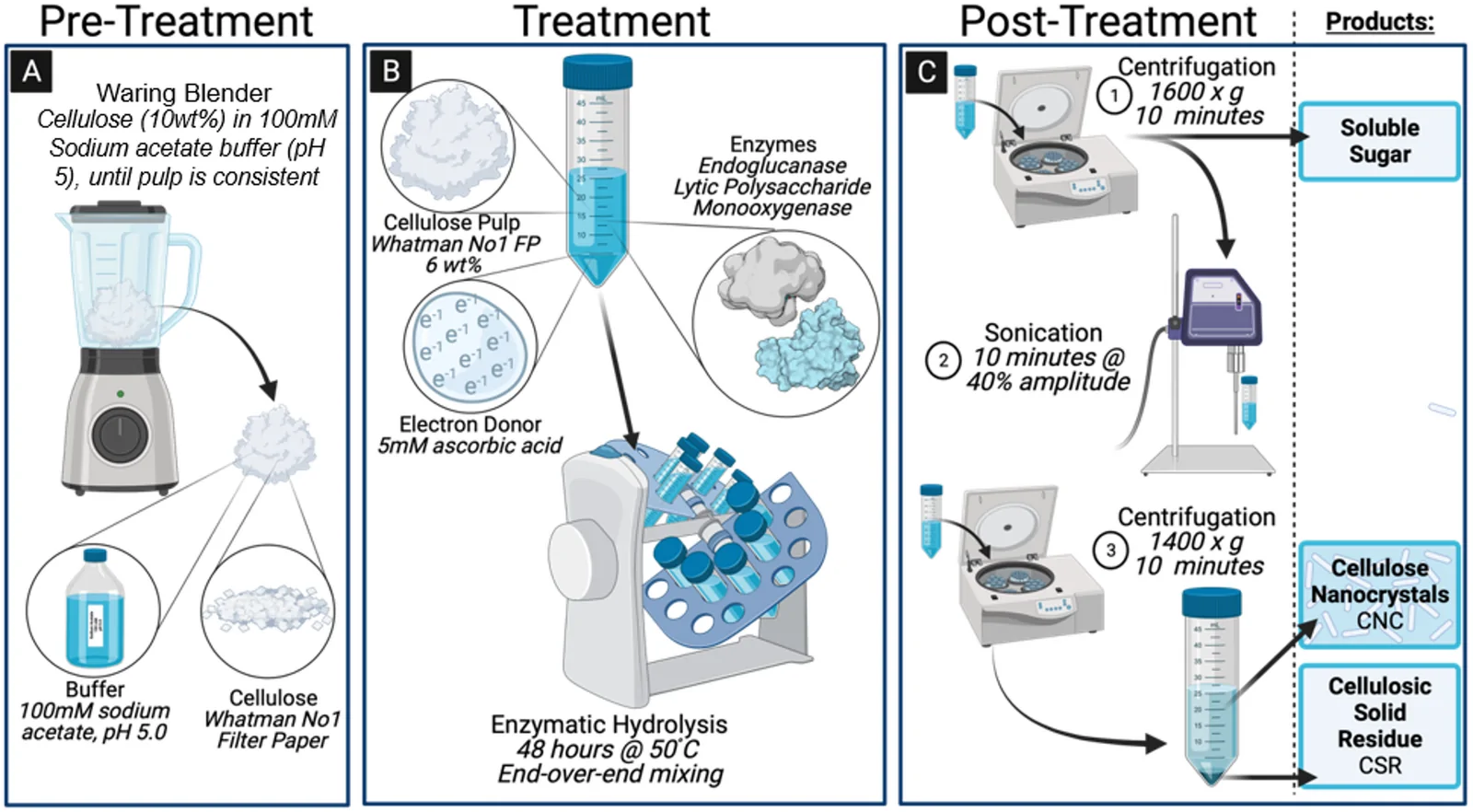
Production and isolation of cellulose nanocrystals from Whatman No.1 filter paper, featuring **A** a blending pretreatment of the cellulose, **B** 48-hour hydrolysis with endoglucanase (NsGH45) and/or lytic polysaccharide monooxygenase *(Ta*AA9A), and **C** a post treatment involving sonication of the treated fibers, and separation by centrifugation into the three products, soluble sugars, CNC, and cellulosic solid residue (CSR). Figure created using BioRender

Filter paper (Whatman No.1, Piscataway, NJ) was used as the model cellulose substrate to enable well-controlled, reproducible results investigating the mechanistic basis of LPMO-endoglucanase synergy. Whatman filter paper is a widely used and well characterized model cellulose with approximate DP ~ 2000 [21] and crystallinity indices of ~ 53 - ~90% [22]. The chemical composition of Whatman No. 1 filter paper is ~ 96% cellulose, ~ 2% hemicellulose, and ~ 0.5% lignin [23]. To prepare the substrate, filter paper was suspended at 10% w/w in 100 mM sodium acetate buffer, pH 5, for approximately 1 h at room temperature before blending in a Waring blender (Fig. 1A). The filter paper slurry was stored in a glass screw cap jar at 4˚C and was brought to room temperature before treating with enzymes. Filter paper was initially prepared as a 10% (w/w) slurry for storage and handling, and then diluted to 6% (w/w) for enzymatic hydrolysis experiments.

### Enzymes and LPMO copper loading

The endoglucanase enzyme (NS 51137) expressed in *Aspergillus oryzae* (referred to as NsGH45 from hereon), the C1/C4 oxidizing *Ta*AA9A LPMO (NS51225) from *Thermoascus aurantiacus*, and *Phanerochaete chrysosporium* cellobiose dehydrogenase (CDH) were provided by Novonesis (Novonesis Inc., Davis, CA USA). NsGH45 was buffer exchanged into 50 mM sodium acetate (pH 5.0) using centrifugal filters (Vivaspin 20, 10 KDa MWCO, Cytiva Life Sciences). *Ta*AA9A was copper saturated by incubating with three-fold molar excess of copper sulfate for 30 min [24–26] followed by desalting in a Zeba Spin™ desalting column (7 K MWCO, 10mL, Thermo Scientific) to remove the excess copper. *Tl*CBH1 from *Trichoderma longibrachiatum* was purchased from Neogen (E-CBH1, Neogen Corporation, Lansing, MI USA) and was buffer exchanged into 50 mM sodium acetate (pH 5.0) using centrifugal filters (Vivaspin 20, 10 KDa MWCO) and stored at 4˚C. Protein concentrations were determined from UV absorption at 280 nm using a NanoDrop™ One Microvolume UV-Vis Spectrophotometer (Thermo Fisher Scientific, Waltman, MA USA).

### Enzymatic hydrolysis of cellulose substrate

Enzymatic hydrolysis experiments were conducted in triplicates using 6% w/w substrate loading in 100 mM sodium acetate buffer, pH 5 in 50 mL conical tubes (Thermo Fisher Scientific, Waltman, MA USA) with a total working mass of 10 g (Fig. 1). Ascorbic acid (5 mM) was used as the electron donor and was freshly prepared before the start of the hydrolysis experiment. The reactions were conducted at 50˚C for 48 h with end-over-end rotation (24 rpm). The selected temperature represents a compromise between maintaining enzyme stability and achieving high catalytic activity. A reaction time of 48 h was chosen to allow the system to approach substrate-limited hydrolysis behavior, where reaction rates plateau due to depletion of accessible cellulose binding sites and evolving interfacial substrate properties. After 48 h, the reactions were terminated by placing the conical tubes in boiling water for 10 min to denature the enzymes.

The first set of hydrolysis experiments used an endoglucanase loading of 3.5 mg NsGH45/g cellulose with the addition of either a high LPMO loading (0.875 mg *Ta*AA9A/g cellulose) or a low LPMO loading (0.35 mg *Ta*AA9A/g cellulose). The second set of hydrolysis experiments maintained an enzyme loading of 3.5 mg protein/g cellulose while varying the molar ratio of *Ta*AA9A-to-NsGH45 from all LPMO to all EG. In both sets of hydrolysis experiments, a no-enzyme control was used to assess the effects of the physical pre- and post-treatments on the cellulosic substrate. The enzyme loading of 3.5 mg/g was chosen to target ~ 0.1 µmole enzyme per gram of substrate, a loading that is expected to be far below substrate-limiting conditions [27]. 

### Post-treatment and isolation of cellulose nanocrystals

The denatured samples were centrifuged at 1600 x g for 10 minutes and approximately 2 mL of supernatant was collected for soluble sugar analysis (Fig. 1C.). The solid residues were then resuspended in Milli-Q water to a final volume of 35mL and sonicated on ice for 10 minutes at 40% amplitude and intermittent 20 s on/10 s off cycles using a probe ultrasonicator fitted with a ¼” tip (Misonix Inc, Farmingdale, NY USA). After sonication, the samples were centrifuged at 1400 x g for 10 min. Cellulose nanocrystals (CNC) were collected in the supernatant, and the pellet (cellulosic solid residue, CSR) was resuspended in Milli-Q to 35mL final volume. The suspension was re-centrifuged, and the supernatant was collected again. The supernatants from both centrifugation steps were combined and used for CNC yield determination. The final masses for both the CNC suspension and the CSR were recorded for yield analysis. Transmission electron microscopy (TEM) images of CNCs obtained from endoglucanase treatments are shown in supplemental information.

The yield (Y) of both the CNC and CSR were calculated according to (Eq. 1)

1 $$\:Y=\frac{{m}_{s}\times\:{c}_{s}}{{m}_{f}}$$

Where *m*_*s*_ is the mass of the CNC or CSR suspension collected (g), *c*_*s*_ is the concentration of the CNC or CSR suspension (g/mL), and *m*_*f*_ is the starting weight of the Whatman filter paper (g). The concentration of the CSR suspension, *c*_*s*_ (%, w.b.), was determined by drying aliquots of the suspension in aluminum weigh pans in an oven at 75˚C until the mass of the dried CSR stabilized (approximately 24 h). The concentration was then calculated by dividing the mass of the dry CSR (*m*_*d*_, g) by the mass of the CSR suspension (*m*_*w*_, g) (Eq. 2).

2 $$\:{c}_{s}=\frac{{m}_{d}}{{m}_{w}}\times\:100\%$$

The concentration of the CNC suspension was determined colorimetrically using an anthrone assay against a glucose standard curve [28].

### Degree of synergy calculations

Degree of synergy is defined as the activity of the mixture of the two enzymes (A_*Ta*AA9A + NsGH45_) divided by the sum of their individual activities (A_*Ta*AA9A_ + A_NsGH45_),

3 $$\:DS=\frac{{A}_{(TaAA9A+NsGH45)}}{{A}_{TaAA9A}+{A}_{NsGH45}}$$

Activity was defined as either the yield of cellulose nanocrystals or the yield of soluble sugar produced. A DS > 1 indicates that the components act synergistically, while a DS < 1 indicates that the two components act anti-synergistically (i.e. impede one another) [29, 30].

### Quantification of solubilized sugars

The soluble sugars (i.e. glucose, cellobiose, and cello-oligosaccharides Glc_3 and_ Glc_4_) released during enzymatic hydrolysis were measured by high performance liquid chromatography using an Agilent 1200-series HPLC with an Aminex HPX-87P column and Micro-Guard deashing and Carbo-P columns (Bio-Rad Laboratories, Inc., Belgium). The sugar concentrations were determined from calibration curves using standard solutions of D-glucose (0.0025–0.5 mg/mL) and cellobiose (0.0025–0.5 mg/mL). The flow rate was 0.6 ml/min at 80˚C using HPLC grade water as the mobile phase.

### Determining the productive *Tl*CBH1 binding capacity of the cellulose nanocrystals and cellulosic solid residue

Productive binding capacity measurements were performed to evaluate how LPMO and endoglucanase treatments alter the availability of accessible sites for processive cellulase action, providing a link between enzymatic modification and substrate accessibility. The productive *Tl*CBH1 binding capacity of the cellulosic solid residue (CSR) from the enzyme treatments (from the hydrolysis with EG and LPMO) was estimated from the maximum hydrolysis rates determined by monitoring the concentration of cellobiose (CB) released over the first seconds ($\:\frac{d\left[CB\right]}{dt}$) (i.e. within the burst phase) of hydrolysis using a cellobiose biosensor (Supplementary Information). Enzyme loadings ranging from 60 to 150 µmole *Tl*CBH1/g of cellulose were added to 0.05 mg/mL of cellulose in a stirred, jacketed cell at 50˚C and 90 rpm to initiate each reaction. Initial cellobiose release rates with respect to enzyme loading were fit to a saturation equation (Eq. 4):

4 $$\:y=\frac{a\:\times\:\:x}{(b+x)}$$

Where *x* is the concentration of *Tl*CBH1 loaded (µmol/g cellulose), *y* is the initial (and maximum) rate of cellobiose release ($\:\frac{d\left[CB\right]}{dt}$), and *a* and *b* are fitting parameters estimating the concentration of total productive binding sites, [S_p_]_t_, and the cellulose affinity parameter, *L*_*m*_, using MATLAB (MATLAB Version 23.2.0.2521687 (R2023b) Update 7, The Mathworks Inc., Massachusetts USA) [31]. From the initial cellobiose release rates, the concentration of productively bound *Tl*CBH1 ([E_BP_]) was estimated, as previously described, from the first order relationship describing the processive hydrolysis of cellulose by a productively bound enzyme with a k_cat_ = 5.3 s^− 1^ at 50˚C (Eq. 5) [27, 32, 33].

5 $$\:\left[{E}_{BP}\right]=\frac{\frac{d\left[CB\right]}{dt}}{{k}_{cat}}$$

The productive binding capacity was estimated as the concentration of productively bound *Tl*CBH1 ([E_BP_]) at saturating conditions, i.e. where every productive binding site is occupied by *Tl*CBH1 and increasing *Tl*CBH1 concentration does not result in increased hydrolysis rates ($\:\frac{d\left[CB\right]}{dt}$).

## Results and discussion

### LPMO aids endoglucanase in cellulose nanocrystal production

To evaluate whether LPMO-EG synergy can promote cellulose nanocrystal (CNC) formation, *Ta*AA9A LPMO and NsGH45 endoglucanase were incubated with pretreated filter paper individually and in combination (Fig. 1). The enzyme loading of NsGH45 endoglucanase was held constant at 3.5 mg EG/g cellulose and *Ta*AA9A LPMO was added at either a high (1:4 EG-LPMO, 0.875 mg LPMO/g cellulose) or low (1:10 EG-to-LPMO, 0.35 mg LPMO/g cellulose) molar loading, hereon referred to as high LPMO and low LPMO.

**Fig. 2:**
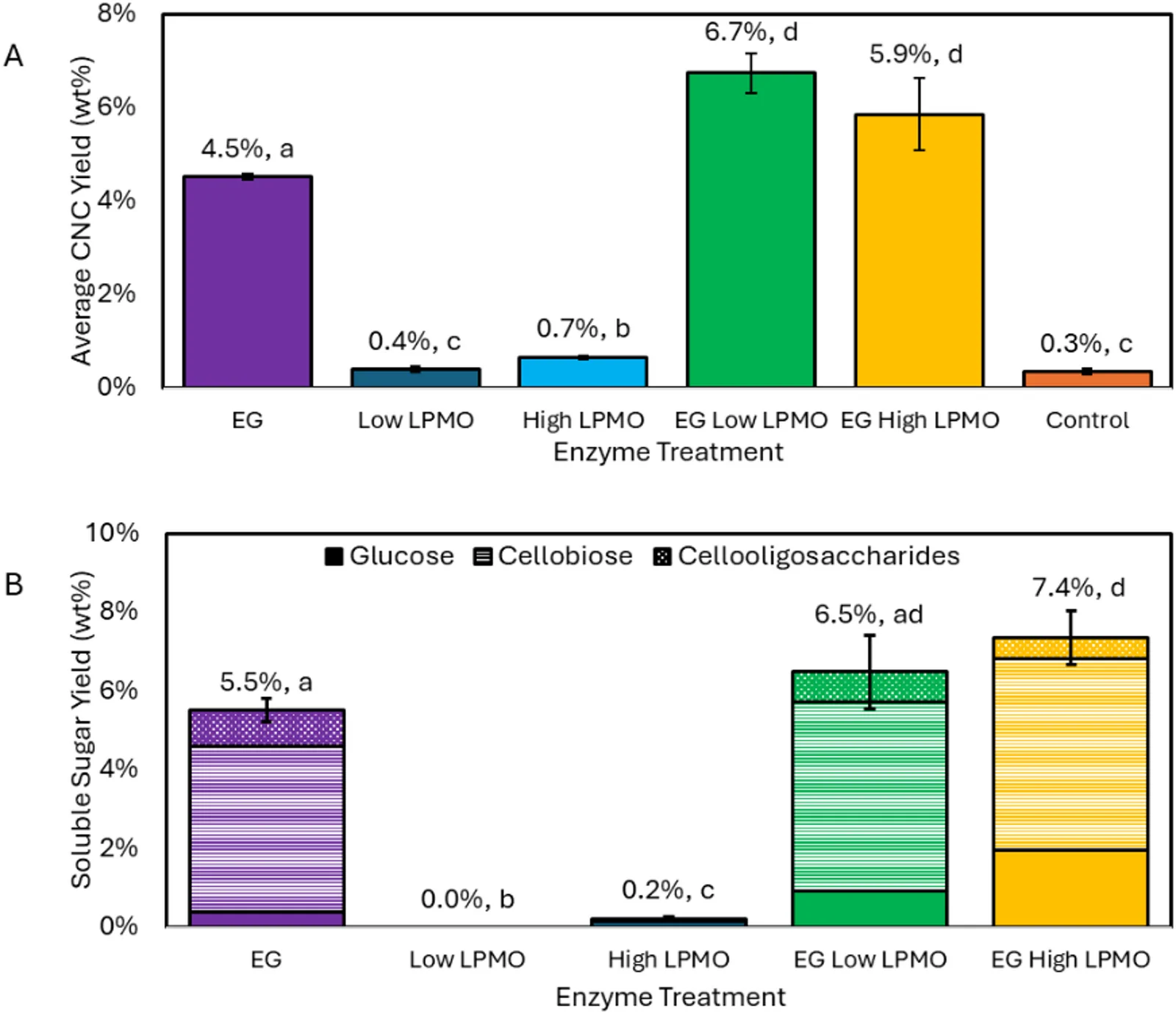
**A** Average CNC yields and **B** Soluble sugar yields and composition from enzyme treatments with *Ta*AA9A LPMO and NsGH45 endoglucanase at 50˚C for 48 h, pH 5.0. Enzyme treatments and loadings were as follows: EG = 3.5 mg NsGH45 endoglucanase/g substrate; Low LPMO = 0.35 mg *Ta*AA9A LPMO/g substrate; High LPMO = 0.875 mg *Ta*AA9A LPMO/g substrate; Control = no enzymes. Error bars represent standard deviation of triplicate experiments (*n* = 3). The same letter indicates no statistically significant difference using a t-test with α = 0.05. In **B**, yields of individual soluble sugars are indicated as follows: Glucose = solid coloring, cellobiose = striped coloring, cellooligosaccharides (cellotriose and cellotetraose) = dotted coloring

An enhanced benefit in cellulose nanocrystal yields was evident when the two enzymes were simultaneously present (Fig. 2A). Treating filter paper with endoglucanase alone liberated 4.5 ± 0.06 wt% of cellulose nanocrystals (Fig. 2A, purple). Minimal CNC yields were observed with LPMO alone at both high and low loading (0.7 ± 0.04 wt% and 0.4 ± 0.20 wt%, respectively) (Fig. 2A, dark blue and light blue). In contrast, coincubation of the two enzymes resulted in clear synergistic enhancement in CNC production. At the low loading of LPMO, CNC yield increased to 6.7 ± 0.42 wt% (DS = 1.37), nearly a 50% increase in cellulose nanocrystal yield. At the high LPMO loading, yields reached 5.9 ± 0.77 wt% (DS = 1.13), a 30% increase. Minimal CNC production in the no enzyme control (0.3 ± 0.07 wt%) confirmed that pre- and post- substrate treatments (blending and sonication) did not significantly contribute to nanocrystal liberation (Fig. 2A, orange).

Soluble sugars released during the enzyme treatments were also analyzed (Fig. 2B). No soluble reducing sugars were detected in the no-enzyme control thereby showing that the physical pre- and post-treatments alone could not release soluble sugars from the substrate and that enzymes were required. LPMO alone produced negligible detectable reducing sugars; oxidized sugars were not analyzed in this study. Coincubation of LPMO with the endoglucanase NsGH45 liberated soluble reducing sugars from the cellulose substrate, demonstrating synergy. Endoglucanase alone released 5.5 ± 0.3 wt% total soluble sugars, majority of which was cellobiose (Fig. 2B). Adding *Ta*AA9A LPMO, at both the high and low loading, resulted in increased concentrations of total soluble sugar: 7.4 ± 0.7 wt% with high *Ta*AA9A loading, and 6.5 ± 0.9 wt% using the low *Ta*AA9A loading.

### LPMO-endoglucanase synergy exhibits divergent optima for CNC production and saccharification

**Fig. 3:**
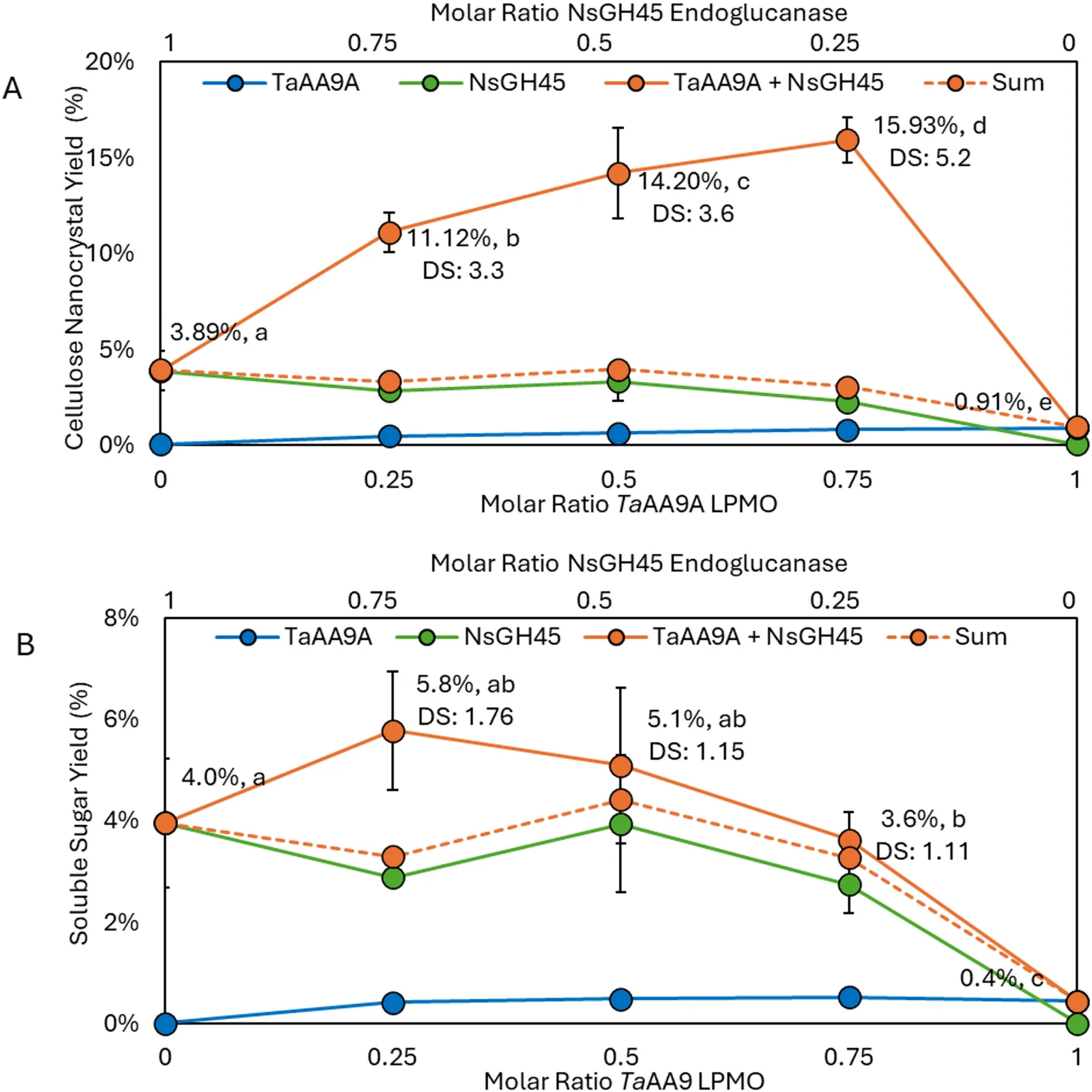
**A** Synergy curve of *Ta*AA9A-NsGH45 mixtures for cellulose nanocrystal yield. Degree of synergy (DS) shows the enhancement in yield from the combination of enzymes compared to the sum of the individual enzymes. **B** Total soluble sugars (cellobiose, glucose, and cello-oligosaccharides) produced during enzyme treatments with varying ratios. In both plots, top x-axes indicate the molar ratio of NsGH45 endoglucanase, ranging from 1 to 0 (left to right), while the bottom x-axes indicate the molar ratio of *Ta*AA9A LPMO, ranging from 0 to 1 (left to right). *Ta*AA9A LPMO loadings ranged from 0 to 3.5 mg/g cellulose. NsGH45 EG loadings ranged from 3.5 to 0 mg/g cellulose. The orange-filled circles with the dotted line indicate the sum of the yields from individual LPMO and EG treatment at that molar ratio. The orange-filled circles with the solid line indicate yields when LPMO and EG were co-incubated at the specified molar ratio. Lines are drawn to guide the eye. Experiments were performed at 50˚C for 48 h at pH 5 and had a total enzyme loading of 3.5 mg protein per gram cellulose. Error bars represent standard deviations from three biological replicates (*n* = 3). The same letter indicates no statistically significant difference using a t-test with α = 0.05

The synergistic interactions between *Ta*AA9A LPMO and NsGH45 EG were more specifically examined by varying their relative molar fractions at a fixed enzyme loading (Fig. 3). Treatment with either enzyme alone resulted in minimal CNC yields; less than 1 ± 0.14 wt% CNC and 3.89 ± 1.0 wt% CNC were liberated using LPMO alone or endoglucanase alone, respectively. Co-incubation of the two enzymes, however, led to substantial increases in CNC production across a broad range of LPMO-endoglucanase ratios. CNC yields increased progressively as the relative proportion of LPMO increased, with a maximum CNC yield of 15.9 ± 1.2 wt% at a 0.25:0.75 EG: LPMO molar ratio (Fig. 3A). This condition also corresponded to the highest degree of synergy (DS = 5.2), indicating that together, LPMO + EG activities were > 5 times more effective at CNC production than when acting separately. The monotonic increase in CNC yield with increasing LPMO fraction suggests that *Ta*AA9A activity plays a dominant role in enabling nanocrystal release, while a minimal but essential level of hydrolytic activity is required to fragment and liberate these crystalline domains.

In contrast to CNC formation, soluble reducing sugar production exhibited a different dependence on enzyme composition (Fig. 3B). Individual enzyme treatments also resulted in low yields, with LPMO producing negligible amounts of soluble sugars and endoglucanase yielding 3.96 ± 1.3 wt%. Upon co-incubation, soluble sugar production increased across all molar ratios, confirming synergistic enhancement; however, the maximum yields (5.8 ± 1.2 wt%) occurred with a composition enriched in endoglucanse (0.75 EG-to-0.25 LPMO). As the reactions shifted from LPMO-rich to endoglucanase-rich ratios, soluble sugar yields increased; indicating that the hydrolytic activity governs the extent of depolymerization with some boost in substrate accessibility. While LPMO contributes to improved overall conversion, higher proportions of endoglucanase drive the reaction toward solubilization rather than retention of insoluble crystalline fragments.

**Fig. 4:**
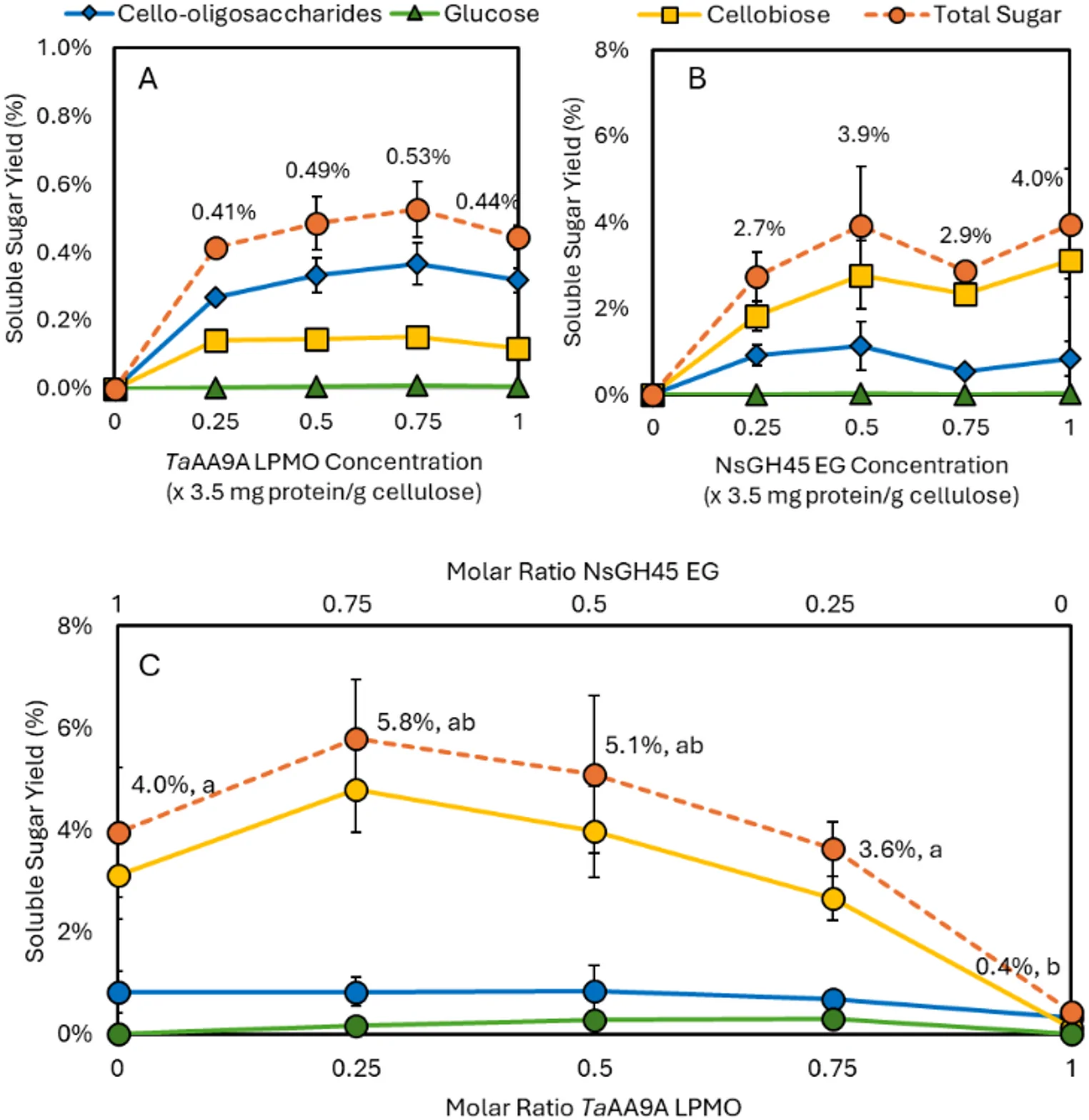
Soluble sugar composition from enzymatic hydrolysis of Whatman No.1 filter paper using **A** *Ta*AA9A LPMO, **B** NsGH45 endoglucanase, and **C** both *Ta*AA9A LPMO and NsGH45 EG. Experiments were conducted at 50˚C for 48 h, pH 5, with the total enzyme loading being 3.5 mg protein/g cellulose. Cello-oligosaccharides (i.e. cellotriose and cellotetraose) yields are represented by the blue-filled circles; cellobiose is represented by the yellow-filled circles, glucose is represented by the green-filled circles, and the sum of the combined soluble sugar yields is represented in orange-filled circles and denoted on the graphs. Lines are drawn to guide the eye. Error bars represent standard deviation from biological replicates (*n* = 3). The same letter indicates no statistically significant difference in cellulose nanocrystal yield at α = 0.05

Together, these trends define a compositional design space in which enzyme ratio governs product selectivity between CNC and soluble sugars (Fig. 3). Within this framework, CNC and soluble sugar trends reveal two distinct but interconnected regimes of cellulose deconstruction. The shape of the synergy curves for both products resembles the classical “endo-exo” behavior observed for enzyme systems with complementary catalytic mechanisms, where maximum enhancement occurs at the intermediate compositions and reflects cooperative interactions (Fig. 4) [30, 34, 35]. In this system, *Ta*AA9A LPMO and NsGH45 endoglucanase both attack internal regions of cellulose but differ in their mode of action, with LPMO targeting highly ordered, crystalline regions [11] and the endoglucanase hydrolyzing less ordered, amorphous domains [36]. The opposing optima for CNC production and sugar release indicate that these enzymes do not simply act additively but instead shift the balance between fragmentation and solubilization depending on their relative ratios.

These results suggest a mechanistic model in which LPMO activity introduces disruptions within the crystalline structure that increase accessibility, enabling endoglucanase to access and cleave previously unavailable regions of cellulose. Under LPMO-rich conditions, this disruption dominates, promoting the release of partially depolymerized crystalline fragments, i.e. CNC. As the ratio of endoglucanase increases, hydrolytic cleavage becomes more extensive, leading to further depolymerization of these fragments into soluble sugars. Thus, CNC production arises from a balance between LPMO-driven accessibility and hydrolysis-driven fragmentation, while sugar production reflects continued hydrolysis beyond fragmentation.

### LPMO activity promotes primary cleavage and enhances endoglucanase activity

The composition of soluble sugar released in the reactions was analyzed to further elucidate contributions of *Ta*AA9A LPMO and NsGH45 endoglucanase and their interactions with cellulose (Fig. 4). The composition of soluble sugar produced varied between the different enzyme treatments suggesting differences in their mechanisms and interactions with the substrate. In the *Ta*AA9A LPMO treatments, longer cello-oligosaccharides (i.e. cellotriose (C3) and cellotetraose (C4)) were the dominant soluble sugar products, followed by smaller amounts of cellobiose and minimal glucose (Fig. 4A). Although the total soluble sugar yield remained very low (< 0.6 wt%) and oxidized products were not quantified, the enrichment of longer oligomers suggest that LPMO activity predominantly results in limited chain cleavage events rather than extensive depolymerization. In contrast, monocomponent endoglucanase treatments produced primarily cellobiose, with smaller contributions from C3 and C4 cello-oligosaccharides and minimal glucose (Fig. 4B), consistent with a hydrolytic mechanism that generates shorter products.

Notably, varying the enzyme loading of either enzyme alone did not significantly affect total sugar yield. This observation suggests that both enzymes rapidly reached a limit imposed by substrate limitations rather than enzyme concentration. In one instance, Angeltveit et al. (2023) showed early curtailment of LPMO activity due to depletion of reductant and consequent enzyme inactivation (37, 38). Similarly, endoglucanase activity has been observed to plateau [39–42], typically associated with the depletion of available productive binding sites [40, 41]. Taken together, similar soluble sugar yields across monocomponent enzyme loadings suggest that the extent of sugar release was most likely limited by substrate availability.

The distinct product distributions observed for the individual enzymes are consistent with differences in catalytic mode. Prior work has shown that cellobiose is the main product of processive hydrolysis, whereas glucose and cellotriose are the primary products released in non-processive primary hydrolysis [12, 29, 43, 44]. The higher abundance of longer cello-oligosaccharides in the reactions with *Ta*AA9A suggest that this LPMO was associated with primary hydrolysis, wherein cleavage of glycosidic bonds generate new chain ends without extensive processive depolymerization [12, 13].

When *Ta*AA9A LPMO and NsGH45 EG were co-incubated, the soluble sugar profile shifted markedly towards cellobiose as the dominant product across all enzyme ratios (Fig. 4C). The concentrations of glucose and cello-oligosaccharides (i.e. cellotriose and cellotetraose) remained relatively constant regardless of the enzyme composition, while cellobiose production increased with increasing endoglucanase fraction, reaching a maximum at 0.75:0.25 EG: LPMO ratio. This shift indicates that the presence of LPMO enhances the extent of hydrolysis carried out by the endoglucanase without fundamentally altering its catalytic mode. Instead, LPMO activity appears to increase the availability of hydrolysable chain segments, thereby enabling more extensive processive hydrolysis and greater cellobiose release by endoglucanases. This finding is consistent with Keller et al., who observed that the addition of LPMO delayed the activity plateau that is typically observed with endoglucanases and speculated that the LPMO produces more attack sites for the endoglucanase [15].

### LPMO activity increases cellulase accessibility to crystalline cellulose

**Fig. 5:**
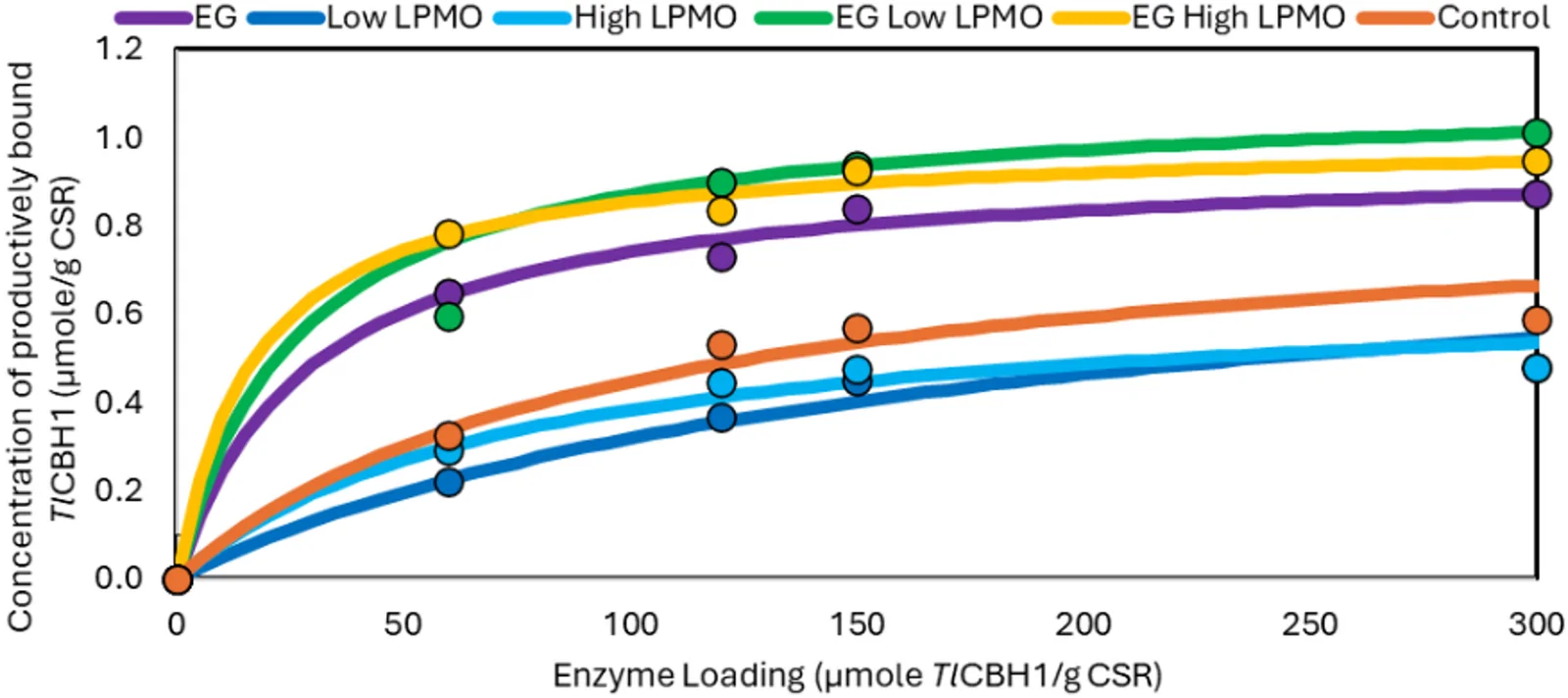
Productive *Tl*CBH1 binding site concentrations (µmol/g) of the cellulosic solid residue remaining after enzymatic treatment and cellulose nanocrystal isolation at various *Tl*CBH1 loadings. Error bars represent standard deviations obtained from three biological replicates (*n* = 3). Lines show curve fits to Eq. 4

Enhanced yields of both CNC and soluble sugars under coincubation conditions suggested that *Ta*AA9A LPMO increases the accessibility of NsGH45 endoglucanase to regions of the cellulosic substrate that are otherwise inaccessible. To examine this hypothesis, the productive cellulase binding capacity was quantified on the cellulosic solid residues following enzymatic treatment. Productive binding sites are locations on the cellulose surface where cellulases can form active complexes to carry out hydrolysis [31, 33]. This study utilized a previously established method to assess the impact of LPMO activity on creating productive binding sites for a cellobiohydrolase (CBH) from *Trichoderma longibrachiatum* (*Tl*CBH1) [13, 31]. Here, the productive binding capacity of a model cellobiohydrolase, *Tl*CBH1 was used as a proxy to assess changes in substrate accessibility due to LPMO and endoglucanase activity (Table 1; Fig. 5).

**Table 1:** Productive *Tl*CBH1 binding capacity of the cellulosic solid resulting from the NsGH45 and *Ta*AA9 treatments. The errors shown indicate 95% confidence intervals

Enzyme Treatment	Enzyme loading (per gram cellulose)	Productive Binding Capacity (µmol/g cellulose)
EG	3.5 mg NsGH45	0.98 ± 0.39
Low LPMO	0.35 mg *Ta*AA9A	0.58 ± 0.32
High LPMO	0.875 mg *Ta*AA9A	0.50 ± 0.10
EG + Low LPMO	3.5 mg NsGH45 + 0.35 mg *Ta*AA9A	1.10 ± 0.03
EG + High LPMO	3.5 mg NsGH45 + 0.875 mg *Ta*AA9A	1.04 ± 0.30
(No Enzyme) Control	0	0.64 ± 0.11

The untreated filter paper substrate exhibited a baseline productive *Tl*CBH1 binding capacity of 0.64 ± 0.11 µmol/g cellulose. Treatment with 3.5 mg/g NsGH45 endoglucanase increased productive *Tl*CBH1 binding capacity to 0.98 ± 0.08 µmol/g cellulose, consistent with the well-established role of endoglucanases in generating new accessible sites for exoglucanases. In contrast, treatment with *Ta*AA9A LPMO alone resulted in a slightly decreased productive *Tl*CBH1 binding capacity to 0.58 ± 0.32 and 0.50 ± 0.10 µmol/g cellulose for the low and high *Ta*AA9A loadings, respectively. This decrease indicates that LPMO activity did not generate productive binding sites for *Tl*CBH1. However, when *Ta*AA9A and NsGH45 were co-incubated with filter paper, productive *Tl*CBH1 binding capacities were boosted by ~ 10% over that of the endoglucanase alone. This synergistic increase in productive binding capacity mirrors enhancements observed in both CNC yield and soluble sugar production, and provide evidence that LPMO activity augments the effectiveness of endoglucanases.

Liquefaction of the cellulose substrate was observed in treatments with NsGH45 endoglucase alone but not in treatments with *Ta*AA9A alone (supplemental information). Liquefaction of the cellulose substrate is well documented with endoglucanase treatments [6, 45]. When the two enzymes were co-incubated with the cellulose substrate, liquefaction was visually more pronounced across conditions. These observations are qualitative and are consistent with enhanced enzymatic disruption of the substrate; however, the present experimental design does not permit quantitative assessment of liquefaction kinetics.

Prior studies have shown that LPMO oxidation promotes cellulose decrystallization by disrupting intermolecular hydrogen bonding in cellulose [12, 13]. Uchiyama et al. observed increased hydrogen bonding between cellulose and water molecules post LPMO-oxidation through their modeling experiments, suggesting that LPMO oxidation allows water to penetrate between cellulose molecules to further disrupt the crystalline structure [12]. This finding was further validated by Angeltveit et al. who found that LPMOs became inactive within 5 h of hydrolysis, but observed that increases in productive *Tl*CBH1 productive binding sites are seen later (24 h) when they speculated that the crystalline structure of cellulose starts to decrystallize and water molecules can penetrate beneath the cellulose molecules, ultimately increasing cellulose accessibility to other cellulases, like exocellulases [13]. We propose that similar synergy is happening in this body of work, where LPMO activity promoted cellulose decrystallization, allowing for increased substrate accessibility by the endoglucanase.

The increase in substrate accessibility for the endoglucanase that results from the LPMO activity was indirectly assessed by measuring productive cellobiohydrolase binding capacity on the cellulosic solid residues. Although alone, *Ta*AA9A LPMO did not increase the accessibility for *Tl*CBH1, a boosted effect on productive binding capacity was observed when co-incubated with NsGH45 LPMO. This suggests that the LPMO disruption allowed for increased cellulose accessibility for the endoglucanase, whose catalytic events resulted in measurable increases in productive binding capacity of *Tl*CBH1. Additionally, increased endoglucanase activity resulted in increased cellulose nanocrystal yields and soluble sugar yields.

While this work focused on product yields and enzyme synergy, the oxidative and hydrolytic mechanisms are expected to influence the physicochemical properties of both the cellulose nanocrystals and residual cellulose. LPMO-mediated oxidation introduces functional groups at the C1 and/or C4 positions, which can alter surface charge, hydration, and fibril–fibril interactions, and has been associated with disruption of crystalline cellulose structure and increased accessibility. In contrast, GH45 endoglucanases typically exhibit limited solubilization but can substantially reduce the degree of polymerization through internal chain cleavage. The combined action of these enzymes is therefore expected to produce CNCs with altered surface chemistry and dispersion behavior, as well as residual cellulose with modified accessibility and structural organization. While these properties were not directly measured in the present study, the observed changes in productive cellulase binding capacity support increased substrate accessibility. Future work will focus on directly quantifying changes in crystallinity, degree of polymerization, and colloidal properties to establish structure–function relationships.

## Conclusions

This study demonstrates that synergy between LPMO (*Ta*AA9A) and endoglucanase (NsGH45) can be harnessed to direct cellulose deconstruction toward either nanomaterial formation or saccharification. In contrast to conventional enzymatic systems that are typically optimized for complete hydrolysis, we show that enzyme composition serves as a key parameter governing product distribution, with LPMO-rich conditions favoring CNC production and endoglucanase-rich conditions promoting soluble sugar release. These findings contribute to the growing body of work exploring enzymatic routes to nanocellulose production as alternatives to acid hydrolysis, which, despite industrial maturity, is associated with environmental and process challenges. By demonstrating enzyme ratio as a tunable process parameter for controlling the balance between fragmentation and solubilization in enzymatic cellulose conversion, this work provides a framework for directing reaction outcomes toward specific product targets without altering operating conditions. Mechanistically, these results support a model in which LPMO activity enhances endoglucanase performance by increasing substrate accessibility, enabling controlled fragmentation of cellulose fibrils while preserving crystalline domains under appropriate conditions.

Looking forward, further work is needed to translate these findings toward practical implementation, including optimizing enzyme loadings, improving CNC yields and process efficiency, and extending these approaches to relevant lignocellulosic substrates. Additionally, future work will extend this framework by quantifying how LPMO and endoglucanase activities influence CNC physicochemical properties, including crystallinity, degree of polymerization, and dispersion behavior. Overall, this work highlights the potential of LPMO-endoglucanase enzyme systems as a flexible platform for the selective production of cellulose-derived materials. By linking enzyme composition to product selectivity, these results provide a foundation for the rational design of sustainable bioprocesses for nanocellulose production.

## Supplementary Information

Below is the link to the electronic supplementary material.

Supplementary Material 1

## Data Availability

Raw data supporting the findings of this study will be made available by the authors upon request.
