# Supplementary material for: Cellulose nanocrystals formation leveraging synergy between lytic polysaccharide monooxygenase and endoglucanase
Source: Bioprocess Biosyst Eng. 2026 Sep 18;49(11):2915–27. doi: 10.1007/s00449-026-03420-9 (PMC13661758; doi:10.1007/s00449-026-03420-9)
Supplement: Supplementary file 1 [file 449_2026_3420_MOESM1_ESM.docx]

Cellulose Nanocrystals Formation Leveraging Synergy between Lytic Polysaccharide Monooxygenase and Endoglucanase

Lauren E Lynn

- Biological and Agricultural Engineering, University of California Davis, Davis, CA USA
- Western Regional Research Center, USDA-ARS, Albany, CA USA

William Hart-Cooper

- Western Regional Research Center, USDA-ARS, Albany, CA USA
- ORCID ID: 0000-0001-8934-1255

William Orts

- Western Regional Research Center, USDA-ARS, Albany, CA USA
- ORCID ID: 0000-0001-7716-7296

Tina Jeoh

- Biological and Agricultural Engineering, University of California Davis, Davis, CA USA
- ORCID ID: 0000-0002-0727-4237
- [tjeoh@ucdavis.edu](mailto:tjeoh@ucdavis.edu)
- Corresponding Author

# S1. Cellulose nanocrystals (CNC) characterization

Cellulose nanocrystals obtained from enzymatic hydrolysis were characterized by measuring degrees of polymerization (DP) and by transmission electron microscopy (TEM) imaging. DP range, obtained from ratios of total glucose concentrations measured by the Anthrone assay and reducing-end concentrations measured by the BCA assay were ~25 ± 1.5 [1]. Representative TEM image of CNCs obtained from endoglucanase treatment are shown in Figure S. 1: TEM images of cellulose nanocrystals produced by endoglucase treatment [1].

Figure S. 1: TEM images of cellulose nanocrystals produced by endoglucase treatment [1].

TEM images were collected as follows - CNC solutions (0.01% in water) were dropped onto 200 mesh Formvar/Carbon coated copper grids, airdried, stained with UranyLess (Electron Microscopy Sciences, Hatfield, PA) for 30 min, and washed over several water drops. Grids were observed in an FEI Tecnai 12 (FEI, Hillsboro, OR) Transmission Electron Microscope at 80 kV. Images were collected using a Gatan Orius SC200 Model 830 camera (Gatan, Pleasanton, CA).

# S2. Cellobiose Biosensor

A three electrode amperometric cellobiose biosensor was used and set up as previously described (30, 31). A platinum wire counter electrode (CE), a reference electrode (RE), and a carbon-paste working electrode (WE) with cellobiose dehydrogenase immobilized on the surface were used to measure the production of cellobiose after the addition of the nonreducing-end specific cellobiohydrolase, *Tl*CBH1 to cellulose. Reactions were conducted in a stirred, jacketed cell held at 50˚C and 90 rpm. Cellobiose dehydrogenase (CDH) working electrodes were prepared using *P. chrysosporium* cellobiose dehydrogenase (CDH) as previously described (30, 31). In brief, graphite powder (Thermo Scientific, Waltham, MA United States), *p*-benzoquinone (Sigma Aldrich), and liquid paraffin (Sigma Aldrich) were thoroughly mixed (approximately 20 minutes) using a mortar and pestle. The resulting paste was packed into a carbon paste electrode (MF-2010, BASi Research Products) and the surface was polished until it was flat and shiny. CDH enzyme solution (4uL, approximately 6 mg/mL) was added to the surface of the polished carbon paste electrode and allowed to dry. The probe was stored overnight in a closed container containing sodium acetate buffer (50mM, pH 5) before being immersed in the buffer the following day. The probe was immersed in the buffer for a minimum of one day before use.

# S3. Cellulose liquefaction

Liquefaction was qualitatively observed from the volume of supernatant post centrifugation using varying enzymes treatments (Figure S. 2). The volume of supernatant increased when *Ta*AA9A LPMO and NsGH45 endoglucanase were coincubated (Figure 4‑9C), compared to the cellulose treated with *Ta*AA9A LPMO (Figure 4‑9A) or NsGH45 EG (Figure 4‑9B) alone. The cellulose treated with *Ta*AA9A LPMO did not liquefy, confirming that endoglucanases are associated with the observed liquefaction. Additionally, cellulose treated with both *Ta*AA9A LPMO and NsGH45 endoglucanase liquefied more rapidly than cellulose treated with only endoglucanase.

Figure S. 2: Cellulose substrate after treatment with (A) TaAA9A LPMO, (B) NsGH45 endoglucanase, and (C) TaAA9A LPMO and NsGH45 endoglucanase to highlight the extent of liquefaction that occurred throughout 48 hour hydrolysis, as seen by the supernatant. Enzyme loadings in each treatment are indicated in the figure.

# S4. References

[1]	L. E. Lynn, “Production of Cellulose Nanocrystals using Enzyme-Assisted Processes,” Ph.D. Dissertation, University of Caifornia, Davis, Davis, California, 2024.

[2]	The CAZypedia Consortium, “Ten years of CAZypedia: a living encyclopedia of carbohydrate active enzymes,” *Glycobiology*, vol. 28, no. 1, doi: doi.org/10.1093/glycob/cwx089.

[3]	A. S. Godoy *et al.*, “Structure, computational and biochemical analysis of PcCel45A endoglucanase from Phanerochaete chrysosporium and catalytic mechanisms of GH45 subfamily C members,” *Sci Rep*, vol. 8, no. 1, p. 3678, Feb. 2018, doi: 10.1038/s41598-018-21798-9.

[4]	W. Yu, I. Mohsin, A. C. Papageorgiou, and D. Li, “Purification and Structural Characterization of the Auxiliary Activity 9 Native Lytic Polysaccharide Monooxygenase from Thermoascus aurantiacus and Identification of Its C1- and C4-Oxidized Reaction Products,” *Catalysts*, vol. 12, no. 2, p. 139, Jan. 2022, doi: 10.3390/catal12020139.

[5]	I. J. Kim, N. Seo, H. J. An, J.-H. Kim, P. V. Harris, and K. H. Kim, “Type-dependent action modes of TtAA9E and TaAA9A acting on cellulose and differently pretreated lignocellulosic substrates,” *Biotechnol Biofuels*, vol. 10, no. 1, p. 46, Dec. 2017, doi: 10.1186/s13068-017-0721-4.

[6]	R. Tokin, J. Ø. Ipsen, P. Westh, and K. S. Johansen, “The synergy between LPMOs and cellulases in enzymatic saccharification of cellulose is both enzyme- and substrate-dependent,” *Biotechnol Lett*, vol. 42, no. 10, pp. 1975–1984, Oct. 2020, doi: 10.1007/s10529-020-02922-0.
